# Clinical, Histological, and Genetic Features of 25 Patients with Autosomal Dominant Progressive External Ophthalmoplegia (ad-PEO)/PEO-Plus Due to *TWNK* Mutations

**DOI:** 10.3390/jcm11010022

**Published:** 2021-12-22

**Authors:** Laura Bermejo-Guerrero, Carlos Pablo de Fuenmayor-Fernández de la Hoz, Pablo Serrano-Lorenzo, Alberto Blázquez-Encinar, Gerardo Gutiérrez-Gutiérrez, Laura Martínez-Vicente, Lucía Galán-Dávila, Jorge García-García, Joaquín Arenas, Nuria Muelas, Aurelio Hernández-Laín, Cristina Domínguez-González, Miguel A. Martín

**Affiliations:** 1 Neuromuscular Unit, Department of Neurology, Hospital Universitario 12 de Octubre, 28041 Madrid, Spain; laura.bermejo@salud.madrid.org (L.B.-G.); carlosdefuenmayor@hotmail.com (C.P.d.F.-F.d.l.H.); 2 Hospital 12 de Octubre Research Institute (imas12), 28041 Madrid, Spain; pabloserra.lor@gmail.com (P.S.-L.); abencinar@hotmail.com (A.B.-E.); joaquin.arenas@salud.madrid.org (J.A.); aurelio.hlain@salud.madrid.org (A.H.-L.); mamcasanueva.imas12@h12o.es (M.A.M.); 3 Biomedical Network Research Centre on Rare Diseases (CIBERER), Instituto de Salud Carlos III, 28029 Madrid, Spain; muelas_nur@gva.es; 4 Mitochondrial Disorders Laboratory, Clinical Biochemistry Department, Hospital Universitario 12 de Octubre, 28041 Madrid, Spain; 5 Department of Neurology, Hospital Universitario Infanta Sofía, 28703 Madrid, Spain; g3.neuro@gmail.com; 6 Department of Neurology, Hospital Universitario Clínico San Carlos, 28040 Madrid, Spain; lmvicente@salud.madrid.org (L.M.-V.); lgaland@yahoo.com (L.G.-D.); 7 Department of Neurology, Complejo Hospitalario Universitario de Albacete, 02006 Albacete, Spain; jorgaga@sescam.jccm.es; 8 Neuromuscular Unit, Department of Neurology, Hospital Universitari I Politècnic La Fe, 46026 Valencia, Spain; 9 Neuromuscular and Ataxias Research Group, Instituto de Investigación Sanitaria La Fe, 46026 Valencia, Spain; 10 Department of Neuropathology, Hospital Universitario 12 de Octubre, 28041 Madrid, Spain

**Keywords:** *TWNK* gene, mitochondrial dysfunction, mtDNA maintenance defects, progressive external ophthalmoplegia

## Abstract

Autosomal dominant mutations in the *TWNK* gene, which encodes a mitochondrial DNA helicase, cause adult-onset progressive external ophthalmoplegia (PEO) and PEO-plus presentations. In this retrospective observational study, we describe clinical and complementary data from 25 PEO patients with mutations in *TWNK* recruited from the Hospital 12 de Octubre Mitochondrial Disorders Laboratory Database. The mean ages of onset and diagnosis were 43 and 63 years, respectively. Family history was positive in 22 patients. Ptosis and PEO (92% and 80%) were the most common findings. Weakness was present in 48%, affecting proximal limbs, neck, and bulbar muscles. Exercise intolerance was present in 28%. Less frequent manifestations were cardiac (24%) and respiratory (4%) involvement, neuropathy (8%), ataxia (4%), and parkinsonism (4%). Only 28% had mild hyperCKemia. All 19 available muscle biopsies showed signs of mitochondrial dysfunction. Ten different *TWNK* mutations were identified, with c.1361T>G (p.Val454Gly) and c.1070G>C (p.Arg357Pro) being the most common. Before definitive genetic confirmation, 56% of patients were misdiagnosed (36% with myasthenia, 20% with oculopharyngeal muscle dystrophy). Accurate differential diagnosis and early confirmation with appropriately chosen complementary studies allow genetic counseling and the avoidance of unnecessary treatments. Thus, mitochondrial myopathies must be considered in PEO/PEO-plus presentations, and particularly, *TWNK* is an important cause when positive family history is present.

## 1. Introduction

Genetic mitochondrial diseases can result from mitochondrial DNA (mtDNA) mutations or from nuclear DNA mutations. Some of the nuclear genes are involved in mtDNA replication and synthesis, and its alteration causes the so-called mitochondrial DNA maintenance defects. The genes in this group encode proteins that play a critical role in different processes: mtDNA replication (*POLG*, *POLG2*, *TWNK*, *TFAM*, *RNASEH1*, *MGME1*, *DNA2*), maintenance of a balanced mitochondrial nucleotide pool (*TK2*, *DGUOK. SUCLG1*, *SUCLA2*, *ABAT*, *RRM2B*, *TYMP*, *SLC25A4*, *AGK*, *MPV17*), and regulation of mitochondrial dynamics (*OPA1*, *MFN2*, *FBXL4*) [1]. Pathogenic mutations in these genes can result in a broad clinical spectrum ranging from severe early-onset disorders to milder presentations with later onset, and are typically linked to the presence of mtDNA depletions and/or multiple mtDNA deletions [1,2].

The *TWNK* gene encodes a replicative mtDNA helicase, thus playing a critical role in the mtDNA replisome. Pathogenic mutations impair its helicase activity and result in replication stalling and impaired mtDNA synthesis [1,2,3].

Autosomal recessive *TWNK* mutations have been linked to infantile-onset spinocerebellar ataxia (IOSCA) and hepatocerebral depletion disorder, whereas dominant *TWNK* mutations are characterized by progressive external ophthalmoplegia (PEO) and ptosis in most patients, typically with an adult-onset presentation. In the latter cases, muscle pathology is associated with multiple mtDNA deletions [1,4]. Although pure-PEO presentations are common, other additional clinical features may be found, such as muscle weakness, exercise intolerance, fatigue, bulbar symptoms, and less frequent cardiac or respiratory involvement, ataxia, neuropathy, cataracts, and parkinsonism, among others [1,4,5,6].

In this study, we summarize the clinical, pathological, and genetic features of 25 patients with confirmed heterozygous mutations in *TWNK* recruited from the Hospital 12 de Octubre Mitochondrial Disorders Laboratory Database, report two novel mutations, and provide a guide for differential diagnosis in clinical practice.

## 2. Materials and Methods

### 2.1. Patients and Data

The 25 study subjects were recruited retrospectively from the database of the Mitochondrial and Neuromuscular Disorders Laboratory of the Hospital Universitario 12 de Octubre in Madrid, a national reference center of rare neuromuscular diseases in Spain. All the patients included in this series were adult patients referred for clinical and/or molecular assessment due to suspected mitochondrial myopathy, identifying pathogenic or likely pathogenic mutations in the *TWNK* gene after genetic testing. We included all patients identified in our database up to March 2021. Partial data of some patients (1, 2, 9, 15, 19, 22, 25) has been published previously [6]. Written informed consent was obtained according to the Ethics Committee requirements.

Clinical evaluations were performed according to the standard of care and were retrospectively reviewed from clinical records.

Circulating GDF-15 was assessed in serum by a quantitative electrochemiluminescence immunoassay in three patients (Elecsys GDF-15; Roche Diagnostics, Basel, Switzerland).

Brain imaging studies were performed with CT in one patient (patient 22) and with MRI (1.5T system) in six patients (patients 2, 3, 4, 5, 7, and 9). Muscle MRI studies were obtained in three patients. Electrophysiological studies were available in sixteen patients. Respiratory function tests were performed when the suggestive clinical data of respiratory involvement was present, being available in three patients. Muscle biopsies were obtained in 19 out of 25 patients and were processed for routine histochemical and immunohistochemical analyses.

### 2.2. Genetic Analysis

Multiple mtDNA deletions were analyzed in skeletal muscle by Southern blot (SB) using mtDNA probes marked with digoxigenin (Roche Diagnostics) and long-range PCR (LR-PCR) of mtDNA using LA Taq DNA Polymerase (Takara Bio, Kusatsu, Japan).

The identification of variants in the *TWNK* gene was performed using intronic primers [7] by direct Sanger sequencing (ABI 3500 Genetic Analyzer, Applied Biosystems, Warrington, UK) or by using a customized next-generation sequencing (NGS) panel of 13 genes associated with defects in mtDNA maintenance (*DGUOK*, *MFN2*, *MPV17*, *OPA1*, *POLG*, *POLG2*, *RRM2B*, *SLC25A4*, *SUCLA2*, *SUCLG1*, *TK2*, *TWNK*, and *TYMP*) and sequencing with the PGM-Ion Torrent platform (Life Technologies, Carlsbad, CA, USA). The alignment of the sequences (ref. CRCh37/hg19) and detection of variants was performed in Torrent Suite (TMAP-variant caller plugin). The annotation and prioritization of variants were carried out by our own scripts integration with Annovar.

Variant prioritization was performed assuming an autosomal dominant or recessive inheritance following the next steps: (i) a Minor Allele Frequency (MAF) < 0.5% in population databases including 1000 Genomes Project [8], Exome Variant Server [9], Genome Aggregation Database (gnomAD) [10], and Collaborative Spanish Variant Server [11]; (ii) intronic variants localized far from 15 nucleotides of the exon/intron junction were discarded; (iii) status and ranking of the variants in the ClinVar database [12]; (iv) variant pathogenicity predictors including Sorting Intolerant from Tolerant (SIFT) [13], PolyPhen-2 [14], MutationTaster [15], Mendelian Clinically Applicable Pathogenicity Score (M-CAP) [16], Protein Variation Effect Analyzer (PROVEAN) [17], and Combined Annotation Dependent Depletion (CADD) Phred [18]; (v) assessment of phylogenetic conservation using Genomic Evolutionary Rate Profiling (GERP) [19], and the Phylogenetic Analysis with Space/Time models (PHAST) programs: phastCons and phyloP [20].

## 3. Results

### 3.1. Clinical Features

**Figure 1:**
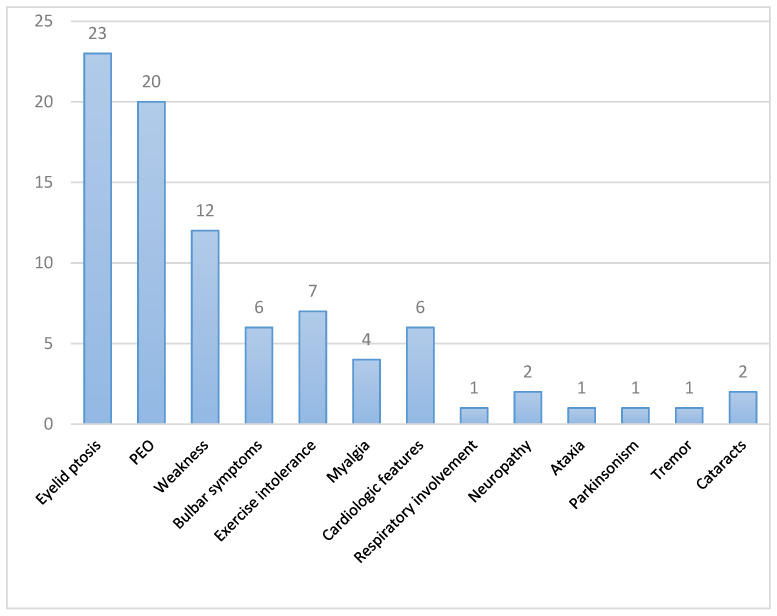
Clinical findings of the 25 patients in our series. Each bar represents the number of patients presenting a specific clinical feature. PEO: progressive external ophthalmoplegia.

The clinical features of the 25 patients from 21 unrelated families are summarized in Table 1 and Figure 1. Sixteen were women (64%). Symptoms started at adulthood (≥18 years old) in 20 patients (80%) and at childhood/adolescence in three patients (12%), being the age at onset uncertain in the two remaining patients. The mean age at onset was 43 years old. The mean age at genetic diagnosis was 63 years (range 28–86 years). Twenty-two patients had first-degree relatives with ptosis and/or ophthalmoplegia.

The most frequent clinical features in our series were ptosis (23 out of 25, 92%) and PEO (20 out of 25, 80%). Five patients reported fluctuating diplopia. Weakness was present in 12 patients (48%), especially affecting the limbs in 11 (44%) patients and the cervical region in 9 patients (36%). Limb weakness was proximal in all cases, with additional distal weakness in one patient; distal weakness without proximal involvement was not reported. Its degree was mild, with no muscular atrophy in any case. Facial weakness was observed in three patients. Seven patients described exercise intolerance (28%), of whom four also presented myalgias (16%). Occasional muscle cramps were reported by two patients, being in one of them concurrent with statin intake and resolved after drug cessation.

Six patients had bulbar involvement (dysphagia and/or dysarthria) (24%), although no patient required enteral nutrition.

Cardiologic abnormalities were observed in six patients (24%), being all mild and unspecific (see Table 1). Similar findings have been reported previously among patients with *TWNK* mutations [4].

Patients with respiratory symptoms were further tested for respiratory muscle dysfunction, confirming in one patient a reduction of the forced vital capacity (FVC) of 57%, and another was eventually diagnosed with obstructive sleep apnea.

Two patients presented features suggestive of polyneuropathy (8%): a 28-year-old man (patient 5) with mildly reduced SNAP amplitude in lower limbs, although asymptomatic, and patient 3 with a highly suggestive clinical picture without electrophysiological tests performed. This latter patient also presented gait ataxia which made walking aid necessary.

Other less common findings observed were parkinsonism and essential tremor, found in one patient each, and two related patients (24 and 25) developed cataracts at the ages of 48 and 50 years with no other known associated risk factors.

Based on the clinical phenotype at initial evaluations, nine patients (36%) were initially misdiagnosed as seronegative myasthenia gravis due to the ocular findings despite the lack of fatigability on examination or other supportive paraclinical data. Three of them, who associated extraocular symptoms, received specific treatment for myasthenia gravis (see Table 1) with no clinical improvement.

Another frequently considered alternative diagnosis before confirmation of *TWNK* mutations was oculopharyngeal muscular dystrophy (OPMD) in five patients (20%).

### 3.2. Complementary Tests

All tests were assessed as standard-of-care clinical procedures. From 18/25 patients with available data, 5/18 patients showed CK levels above the upper limit of the reference (28%), with a mean value of 278 U/L.

Markers related to mitochondrial dysfunction were also studied, such as lactate and the more specific and sensitive GDF-15, the latter only recently available in our center. Mild hyperlactacidemia was found in three out of six tested patients (3.24–3.7 mmol/L). Serum GDF-15 levels could be determined only in 3/25 patients, being elevated in two (1454 pg/mL in patient 5 and 2727 pg/mL in patient 6) and normal in one (991 pg/mL in patient 2). Results were adjusted according to age. Patient 2, unlike the others, did not have clinical data of muscular involvement additional to PEO.

Electrophysiologic testing, performed in 15/25 patients, showed myopathic features on needle EMG testing in nine (60%), neurogenic features in nerve conduction studies and needle EMG in two (13%), and was normal in four patients (27%).

Muscle MRI was performed in 3/25 patients with extraocular complaints, with normal results in one patient and an unspecific pattern of muscle edema and fatty infiltration in the other two (see Table 2). Brain imaging (MRI or CT) was performed in 7/25 patients, with normal results or mild incidental findings displayed in all of them.

Muscle biopsy was available in 19/25 patients, with signs of mitochondrial dysfunction (RRF or COX-negative fibers) presenting in all of them. Four patients who did not undergo biopsy were relatives of others with confirmed *TWNK* mutations.

Multiple mtDNA deletions were found in the skeletal muscle of all patients tested (17/17).

### 3.3. Genetic Results

**Figure 2:**
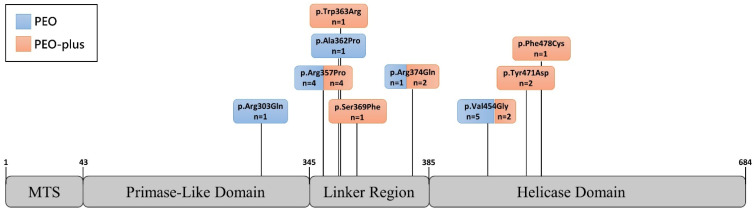
Phenotypic features depending on the affected protein domain. TWNK protein representation showing the distribution of the mutations identified in our cohort along the domains of the protein and the number of patients presenting PEO (blue) or PEO-plus (orange) phenotype for each variant. PEO: progressive external ophthalmoplegia. MTS: mitochondrial targeting sequence. Numbers on the TWNK protein denote the aminoacidic residue delimiting the domains.

Ten different mutations in the *TWNK* gene were identified, all of them being heterozygous missense variants clustered in exons 1 and 2. The most frequent mutations identified in our series were c.1361T>G (p.Val454Gly), found in seven patients from six families, and c.1070G>C (p.Arg357Pro), identified in seven patients from five families (see Table 2). Other *TWNK* mutations identified were c.1121G>A (p.Arg374Gln) in three unrelated patients (12%), c.1411T>G (p.Tyr471Asp) in two siblings (8%), and c.908G>A (p.Arg303Gln), c.1433T>G (p.Phe478Cys), c.1084G>C (p.Ala362Pro), c.1071G>C (p.Arg357Pro), c.1087T>A (p.Trp363Arg), and c.1106C>T (p.Ser369Phe) in single cases. Figure 2 shows the location of each mutation in the gene.

The last two have not been previously reported. Similarly to other known mutations, both are missense variants located at exon 1 of the *TWNK* gene, and affect linker region in the protein product [2,4]. They are predicted to be pathogenic according to ACMG classification [21], as they both fulfil three criteria of moderate pathogenicity, which are: (1) occurrence at a mutational hot spot, (2) absence from control subjects in Genome Aggregation Database (gnomAD), Exome Sequencing Project, or 1000 Genomes Project, and (3) being a missense change at an amino acid residue where a different pathogenic missense changes have already been identified before, such as c.1088G>T (p.Trp363Leu), c.1105T>C (p.Ser369Pro), and c.1106C>A (p.Ser369Tyr) [21]. Additional supportive evidence for their pathogenicity is the low rate of benign missense variants in the *TWNK* gene, and in the case of c.1106C>T the presence of multiple predictors supporting a deleterious effect (such as Bayes Del_addAF, DANN, DEOGEN2, EIGEN, FATHMM-MKL, LIST-S2, M-CAP, MVP, Mutation Assessor, MutationTaster and SIFT, with no benign predictions).

## 4. Discussion

Our study describes the clinical and molecular characteristics of 25 adult patients with genetically confirmed autosomal dominant *TWNK* mutations.

The predominant clinical features among our patients were ptosis (92%) and PEO (80%). This is consistent with descriptions in previous works about *TWNK*-related adPEO, where PEO and ptosis are almost universal findings [1,4,5,7], with ptosis often being the first sign and preceding the ocular weakness [5,22].

The presence of PEO has been identified as a clinical hallmark of mtDNA disorders since the earliest clinical descriptions of mitochondrial syndromes [22,23], which was confirmed by subsequent studies. In this sense, the most frequent underlying defects in PEO patients are sporadic single large-scale mtDNA deletions [6,22], followed by mutations in mtDNA maintenance nuclear genes, which are the most common genetic finding when positive family history is present [6,23]. Recent series of PEO patients state that among these latter disorders, *POLG* and *TWNK* mutations are the most frequently involved nuclear genes with a similar rate of occurrence [6,24]. Although, in general terms, *POLG* has been commonly linked to a PEO-plus phenotype and *TWNK* to pure-PEO [1,4,6], additional associated features in *TWNK* patients are not rare, being the PEO-plus phenotype more common in our cohort, as in some other previously reported works [5].

The presence of associated proximal muscle weakness is common in all series published to date, ranging between 30 and 80% [3,4,5]. Other possible associated symptoms described in the literature include bulbar symptoms (12–31%), respiratory involvement (17%), cardiological abnormalities (11–24%), polyneuropathy (15–40%), ataxia (9%), parkinsonism, and tremor (13%), and less frequently cataracts, visual disturbances and sensorineural hearing loss. All of them were present in our series in a similar frequency [3,4,5].

With respect to cardiac manifestations, it has been proposed that myocardial involvement and arrhythmias could be part of the TWNK phenotype [4]. We also found unspecific and mild cardiac abnormalities in about a quarter of our patients, but it is unclear if *TWNK* mutations play a causative role in their development.

Regarding the diagnostic approach, it is worth noting that although not in all cases, while myopathic changes were identified in the electrophysiological studies, it did allow us to rule out signs of alteration of the neuromuscular junction, supporting its utility in the differential diagnosis in patients with ocular symptoms. On the other hand, plasma CK and lactate levels found in our series would fit in the previously reported ranges of hyperCKemia and hyperlactacidemia in patients with mitochondrial myopathy [3,6,23]. Thus, CK levels in patients harboring *TWNK* mutations are typically normal or only slightly elevated. An exception among mitochondrial myopathies is a TK2 deficiency, where hyperCKemia is common and typically 10-fold above the upper normal limit (up to 30-fold) [25,26,27]. Therefore, these two parameters lack enough sensitivity and specificity for the diagnosis of mitochondrial diseases [22,26,28]. Although GDF15 serum levels seem to provide a better prediction of mitochondrial disease regardless of clinical phenotype (with a diagnostic sensitivity of around 80% [28,29,30]), the small number of patients evaluated in our series prevents us from drawing conclusions about its diagnostic performance in patients with PEO syndrome. Finally, when a muscle biopsy was performed, it allowed guiding the genetic study in all cases, since signs of mitochondrial dysfunction and mtDNA multiple deletions were found in all of them.

Of the over 40 known adPEO-causing *TWNK* mutations, almost all are missense changes, occurring throughout the entire length of the gene but clustering at exons 1 and 2, specially affecting the primase-like and linker region protein domains [2,4,24]. Our findings are consistent with this, with the two novel likely pathogenic identified variants, c.1087T>A (p.Trp363Arg) and c.1106C>T (p.Ser369Phe), being missense changes located at exon 1 in the linker region. In our cohort, we did not identify phenotype-genotype correlations since the phenotype did not depend on the affected protein domain (see Figure 2).

Among our patients, we observed frequent misdiagnosis in the form of seronegative myasthenia gravis and OPMD, which should be kept in mind for the differential diagnosis, as they can present with similar clinical findings.

Autoimmune myasthenia gravis with prominent ocular involvement may be confused with PEO, although its clinical presentation and course are different: myasthenia presentation is typically acute or subacute, with marked diurnal fluctuations and fatigability, and variable measurements on examination. On the other hand, PEO presents typically with chronic and more constant findings. Although PEO may show subtle eyelid fatigability, marked fatigue on prolonged maintenance of a certain position or highly variable measurements on a given examination strongly supports the diagnosis of myasthenia gravis. The same applies to muscular weakness at limbs and other territories [22]. Furthermore, additional neurological features possibly associated to *TWNK* phenotype, as well as the existence of positive family history, are absent in autoimmune myasthenia. In those cases where the clinical picture does not allow clear distinction, an electrophysiologic test, serum antibodies determination and a treatment trial may help, bearing in mind that an absolute lack of response to prednisone suggests an alternative diagnosis other than myasthenia gravis. However, single-fiber EMG can be confusing, as CPEO in mitochondrial myopathies may also show an abnormal jitter [22,31].

Differentiation from congenital myasthenic syndromes can be more challenging. These are very rare inherited diseases caused by mutations in genes encoding for different proteins expressed at the neuromuscular junction that present with a widely heterogeneous clinical picture. Typically, they start within the first years of life with fatigable weakness most commonly ocular and other cranial muscles, as well as limbs and respiratory muscles, with different patterns and severity [32,33]. However, there are some cases that are easier to confuse with mitochondrial myopathies due to the existence of fixed weakness and a lack of clear fatigability upon examination. In some cases, there may even be an increase in plasma CK levels and negative COX fibers in the muscle biopsy. [34]. In these cases, a key finding for diagnosis is the presence of abnormal repetitive stimulation in the electrophysiological test, which is not seen in mitochondrial myopathies [31]. Genetic confirmation will provide the definitive diagnosis.

OPMD is a dominantly inherited disorder caused by the expansion of GCN triplets in the *PABPN1* gene, usually with a late onset throughout the fifth or sixth decade of life. It initially presents with ptosis and dysphagia and, as the disease progresses, it can affect other muscles, mainly the proximal limb muscles [35]. For the differential diagnosis with mitochondrial myopathies, a muscle biopsy can be helpful showing dystrophic changes and rimmed vacuoles, although some can associate slight mitochondrial alterations [36]. Importantly, muscle MRI has also been proven useful, as OPMD has a characteristic pattern of muscle involvement consisting of an early combination of fat replacement in the tongue, adductor magnus, and soleus [37]. Performing it early in the diagnostic process can help direct genetic testing and avoid muscle biopsy.

**Figure 3:**
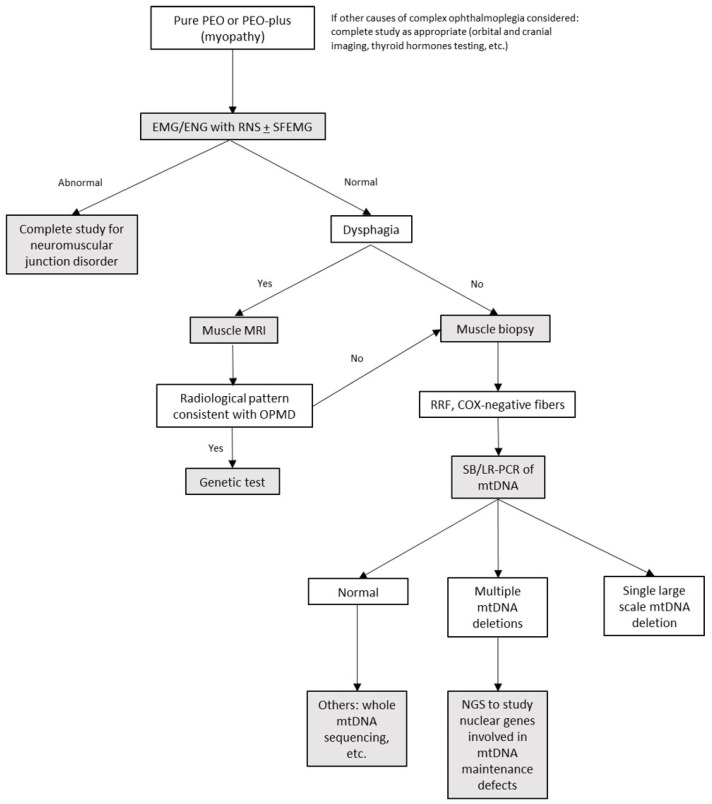
Proposed algorithm for differential diagnosis. Abbreviations: LR-PCR: long-range Polymerase Chain Reaction, NGS: next generation sequencing, RNS: repetitive nerve stimulation, SB: southern blot, SFEMG: single-fiber electromyography.

We propose the following approach for the differential diagnosis of patients manifesting with chronic PEO (see algorithm in Figure 3): in addition to a conventional EMG, it would be of interest to perform a repetitive nerve stimulation test in patients with suspicion of a neuromuscular junction disorder, because it has a high positive predictive value. However, its sensitivity is around 70% in generalized myasthenia, and notably lower in ocular myasthenia (20–50%) [38], in which an ice pack test and a treatment trial may help to confirm the diagnosis if fatigability is found on examination. When a muscle disorder is suspected, a muscle biopsy (which could be obtained with a minimally invasive procedure, such as a needle biopsy) allows us to guide genetic diagnosis (ragged-red COX negative fibers vs. rimmed vacuoles and dystrophic changes in mitochondrial myopathies and OPMD respectively), but also to obtain muscle mitochondrial DNA and look for single or multiple mtDNA deletions, thus identifying primary mtDNA disorders and allowing a more precise selection of patients for additional nuclear DNA analysis when necessary. In patients with limb weakness, prior to muscle biopsy, a muscle MRI can distinguish patients with OPMD who will be diagnosed with targeted analysis of the *PABPN1* gene.

## 5. Conclusions

This work provides a detailed description of the clinical and paraclinical characteristics of patients with dominant *TWNK* mutations manifesting with PEO phenotype. We also provide a differential diagnosis approach to minimize the diagnostic odyssey and avoid unnecessary and potentially harmful treatments in these patients.

## Figures and Tables

**Table 1:** Clinical features of patients with autosomal dominant progressive external ophthalmoplegia and *TWNK* mutations.

Patient	Sex	Age (y.o)	Age at Onset	Family History	Eyelid Ptosis	CPEO	Weakness	Dysarthria/Dysphagia	Cardiac/Respiratory Involvement	Others	Previous Diagnosis and Treatment
1	M	65	30	Yes. Nephew of pt. 23	Yes	Yes	Inferior facial, mild cervical, proximal UL and LL	Dysarthria and dysphagia	No suggestive clinical data; normal ECG, FVC 105%	No	OPMD; no treatment
2	F	74	Adult	Yes	Yes	Yes	No	No	Tachycardia under study, ECG: LAFB	No	Seronegative ocular MG, no treatment
3	M	82	40	Yes Brother of pt. 7	Yes	Yes	Mild cervical, mild proximal UL and LL	No	No suggestive clinical data	Exercise intolerance, ataxia, PNP	Seronegative MG; pyridostigmine (NR)
4	F	79	60	Yes	yes	Yes	Mild cervical, mild proximal LL	Dysphagia	Mild LVH, normal ECG; sleep apnea with CPAP indication	Myalgias, exercise intolerance	Seronegative MG; pyridostigmine and GC (NR)
5	M	28	17	No	Yes	Yes	No	No	Palpitations with normal TTE and Holter-ECG	Myalgias, exercise intolerance, subclinical PNP	Seronegative MG; no treatment
6	F	64	60	Yes	Yes	Yes	No	No	No suggestive clinical data	Episodes of acute worsening with bulbar and limb weakness	Seronegative MG; pyridostigmine (NR), acute episodes: IVIg, plasma exchange, IS (NR)
7	F	86	60	Yes Sister of pt. 3	Yes	No	Mild proximal LL	No	Suspicion of sleep apnea; mild LVEF reduction	Essential tremor (UL, head and jaw), exercise intolerance	-
8	M	68	30	Yes	Yes	Yes	No	No	No suggestive clinical data	-	-
9	M	36	25	No	Yes	Yes	No	No	No suggestive clinical data; ECG: RBBB, normal TTE	-	Seronegative MG, OPMD; no treatment
10	F	58	50	Yes	Yes	Yes	No	No	No suggestive clinical data	-	-
11	F	59	43	Sister with epilepsy. Both parents dementia	No	No	Mild cervical, mild proximal UL and LL	No	No suggestive clinical data	Generalized myalgias at rest, cramps with statins in the past, exercise intolerance	-
12	M	67	Adult onset	Yes	Yes	Yes	No	No	No suggestive clinical data	-	OPMD; no treatment.
13	F	72	40	Yes	Yes	No	Mild cervical	Dysphagia	No suggestive clinical data	-	OPMD, no treatment
14	M	54	40	Yes	Yes	No	No	No	No suggestive clinical data	-	Seronegative MG.
15	F	51	Not reported	Yes	No	Yes	No	No	No suggestive clinical data	-	Seronegative MG
16	F	67	Adult onset	Yes	Yes	Yes	Proximal UL and LL	No	No suggestive clinical data	-	OPMD; no treatment.
17	F	32	Not reported	Yes	Yes	Yes	Cervical, proximal UL and LL	No	No suggestive clinical data	Exercise intolerance	-
18	F	49	14	Yes	Yes	Yes	Cervical, proximal and distal UL and LL	Dysphagia	No suggestive clinical data	-	-
19	F	44	Childhood onset	Yes	Yes	Yes	No	No	No suggestive clinical data	Occasional cramps; fatigable ocular weakness reported	Seronegative MG
20	F	78	50	Yes	Yes	Yes	Proximal at limbs	No	No suggestive clinical data	-	-
21	M	80	45	Yes Brother of pt. 22	Yes	Yes	Orbicularis oculi, inferior facial, cervical, proximal at limbs	Dysarthria and dysphagia	No suggestive clinical data; FVC 100.9%; mild LVH	Myalgias, exercise intolerance	-
22	F	80	40	Yes Sister of pt. 21	Yes	Yes	Orbicularis oculi, cervical, limbs	Dysphagia	FVC 57%	Parkinsonism	-
23	F	82	60	Yes Aunt of pt. 1	Yes	No	No	No	Not reported	-	-
24	M	55	48	Yes Son of pt. 25	Yes	Yes	No	No	Not reported	Cataracts 48 y.o	-
25	F	76	64	Yes Mother of pt. 24	Yes	Yes	No	No	Not reported	Cataracts 50 y.o	-

CPEO: chronic progressive external ophthalmoplegia; ECG: electrocardiogram; ENG: electroneurography; FVC: forced vital capacity; GC: glucocorticoids; IS: immunosuppression; IVIg: intravenous immunoglobulins; MG: myasthenia gravis; LAFB: left anterior fascicular block; LL: lower limbs; LVEF: left ventricular ejection fraction; LVH: left ventricular hypertrophy; NR: no response; OPMD: oculopharyngeal muscular dystrophy; PNP: polyneuropathy; RBBB: right bundle branch block; RI: respiratory insufficiency; TTE: transthoracic echocardiography; UL: upper limbs; y.o: years old.

**Table 2:** Complementary studies.

Patient	CK (IU/L)	Lactate (mmol/L)	GDF15 (pg/mL)	EMG/ENG	Muscle MRI	Muscle Biopsy	TWNK Variant	Protein Change	Multiple mtDNA Deletions (Skeletal Muscle)
1	300	-	-	-	-	-	c.1361T>G	p.Val454Gly	-
2			990.6	Normal		RRF and COX negative fibers	c.1070G>C	p.Arg357Pro	Y
3	68	3.7	-	-	-	-	c.1070G>C	p.Arg357Pro	-
4	21	-	-	Normal	IL: diffuse edema of both gastrocnemius and lateral and distal portion of left soleus	Frequent RBF, 1% COX negative fibers	c.1070G>C	p.Arg357Pro	Y
5	101	1.7	1454	Mild SNAP amplitude reduction in both surals and superficial peroneals	IL: normal	9% COX negative fibers	c.1121G>A	p.Arg374Gln	Y
6	37	-	2727	-	-	RRFs and 1% COX negative fibers.	c.1361T>G	p.Val454Gly	-
7	300	-	-	Myopathic	-	RRF, COX negative fibers, complex IV deficiency	c.1070G>C	p.Arg357Pro	Y
8	Normal	-	-	Myopathic	-	Occasional RRF	c.1361T>G	p.Val454Gly	Y
9	305	Normal	-	Normal	-	Frequent RBF (most of them COX negative, 3.5% of total fibers)	c.1121G>A	p.Arg374Gln	Y
10	-	-	-	-	-	-	c.1361T>G	p.Val454Gly	-
11	224	-	-	Moderate chronic neurogenic signs R L5	-	Frequent subsarcolemic SDH accumulation, frequent COX negative fibers, type I fiber predominance	c.1087T>A (new description)	p.Trp363Arg	Y
12	-	-	-	Moderate myopathic signs	-	Unspecific: isolated RBF, <1% COX negative fibers	c.908G>A	p.Arg303Gln	Y
13	-	-	-	-	Diffuse fatty infiltration of paravertebral muscles, abnormal signal at both serratus anterior	RRF and frequent COX negative fibers	c.1106C>T (new description)	p.Ser369Phe	Y
14	95	-	-	Myopathic	-	RRF, 10% COX negative, type 1 fiber predominance	c.1070G>C	p.Arg357Pro	Y
15	Normal	-	-	-	-	Occasional RRF, 2% COX negative fibers	c.1361T>G	p.Val454Gly	Y
16	74	-	-	Myopathic	-	Unspecific oxidative alterations, 3 RRF	c.1361T>G	p.Val454Gly	Y
17	-	-	-	-	-	RRF, frequent COX negative fibers, some internal nuclei	c.1121 G>A	p.Arg374Gln	Y
18	-	-	-	-	-	-	c.1433T>G	p.Phe478Cys	-
19	312	-	-	Myopathic	-	3% COX negative fibers	c.1084G>C	p.Arg362Pro	Y
20	Normal	Normal	-	Myopathic	-	RRF and COX negative fibers	c.1071G>C	p.Arg357Pro	Y
21	113	Raised	-	Myopathic	-	RRF, massive mitochondrial accumulations with SDH, loss of COX activity. Complex I and IV deficiency	c.1411T>G	p.Tyr471Asp	Y
22	66	-	-	-	-	-	c.1411T>G	p.Tyr471Asp	-
23	54	-	-	Myopathic	-	RRF, type 1 fiber predominance	c.1361T>G	p.Val454Gly	-
24	-	-	-	-	-	-	c.1070G>C	p.Arg357Pro	-
25	61	3.24	-	Normal ENG	-	RRF	c.1070G>C	p.Arg357Pro	Y

NA: not available; RBF: ragged blue fibers; RRF: ragged red fibers; SVD: small vessel disease; Y: Yes; -: not performed or not data recorded.

## Data Availability

The datasets analyzed during the current study are available from the corresponding author upon reasonable request.
